# Effect of Cry Toxins on *Xylotrechus arvicola* (Coleoptera: Cerambycidae) Larvae

**DOI:** 10.3390/insects13010027

**Published:** 2021-12-26

**Authors:** Álvaro Rodríguez-González, Alejandra J. Porteous-Álvarez, Marcos Guerra, Óscar González-López, Pedro A. Casquero, Baltasar Escriche

**Affiliations:** 1 Grupo Universitario de Investigación en Ingeniería y Agricultura Sostenible (GUIIAS), Instituto de Medio Ambiente Recursos Naturales y Biodiversidad (INMARENBIO), Escuela de Ingeniería Agraria y Forestal (EIAF), Universidad de León, 24071 Leon, Spain; apora@unileon.es (A.J.P.-Á.); pacasl@unileon.es (P.A.C.); 2 Grupo Universitario de Investigación en Ingeniería y Agricultura Sostenible (GUIIAS), Escuela de Ingeniería Agraria y Forestal (EIAF), Campus de Ponferrada, Universidad de León, 24401 Ponferrada, Spain; mgues@unileon.es; 3 Departamento de Agricultura y Alimentación, Complejo Científico Tecnológico, Universidad de La Rioja, Área de Producción Vegetal, 26006 Logrono, Spain; oscargonzalezl@unirioja.es; 4 Instituto Universitario de Biotecnología y Biomedicina (BIOTECMED), Departamento de Genética, Universitat de Valencia, 46100 Burjassot, Spain

**Keywords:** vineyards, insect pest, Xylophagous polyphagous, *Bacillus thuringiensis*, crystal proteins

## Abstract

**Simple Summary:**

*Xylotrechus arvicola* is a destructive pest in vineyards (*Vitis vinifera*) in the main wine-producing areas of the Iberian Peninsula. *X. arvicola* larvae bore into the grapevine wood-making galleries, thus damaging the plant both directly and indirectly. The susceptibility of *X. arvicola* larvae to five coleopteran toxic Cry proteins was evaluated under laboratory conditions in order to deepen the knowledge of the effect of these proteins on this insect throughout its biological development. The Cry proteins tested could be applied to control *X. arvicola* larvae since they were able to kill them and cause serious alterations in the larvae during the remaining months of development that followed. The data presented suggest that these Cry proteins can be used as bioinsecticides against the larvae of this insect, in order to avoid the rapid evolution of resistance against these toxins since not all of the larvae were killed and thus increase vine wood protection.

**Abstract:**

The beetle *Xylotrechus arvicola* is a destructive pest in vineyards (*Vitis vinifera*) in the main wine-producing areas of the Iberian Peninsula. *X. arvicola* larvae bore into the grapevine wood-making galleries, thus damaging the plant both directly and indirectly; the latter through the proliferation of wood fungi, which can invade the inside of the plant, decreasing the quality and quantity of its production. The susceptibility of *X. arvicola* larvae to five coleopteran toxic Cry proteins (Cry1B, Cry1I, Cry3A, Cry7A, and Cry23/37) was evaluated under laboratory conditions in order to deepen the knowledge of the effect of these proteins on this insect throughout its biological development. Cry7Ab and Cry1Ba were the most effective in controlling *X. arvicola* larvae due to the significant reduction in larvae survival (32.9 and 25.9 days, respectively), and by causing serious alterations in the larvae during the remaining months of their development. The developmental stage of the prepupal and pupal stages was not affected by the previous ingestion of Cry proteins. The Cry proteins tested could be applied to control *X. arvicola* larvae since they were able to kill them and cause serious alterations in the larvae during the remaining months of development that followed. The data presented suggest that these Cry proteins can be used as bioinsecticides against the larvae of this insect, applying them only at the moment when the larvae hatch from the egg outside the grapevine wood (this would only be useful and justified if the economic threshold is exceeded) in order to avoid the rapid evolution of resistance against these toxins since not all of the larvae were killed and thus increase vine wood protection.

## 1. Introduction

Cerambycidae is one of the largest families of Coleoptera [1,2]. Cerambycid beetles can be found worldwide [3]. Cerambycids are phytophagous (larvae, round-headed borers, usually burrowing in the tissues of woody plants in conditions ranging from alive to moribund, to dead and decomposing). Many species are important pests in forests, plantations, and even trees in urban environments [4].

Cerambycid pests generate serious economic losses in industrial wood production worldwide [5,6,7]. *Vesperus xatarti* (Dufour-Mulsant), which is the most prominent among the cerambycids that attack *Vitis vinifera* wood in vineyards [8], has been recognised as an insect pest since the mid-19th century [9], and shortly after was recorded in Spanish vineyards [10,11]. *Clytus arietis* (L.) has been reported as a pest in Spanish [11,12] and French vineyards [13]. Around the world, *Acalolepta vastator* (Newman) has caused severe damage to Australian vineyards [14], whereas *Xylotrechus pyrrhoderus* (Bates) has caused severe economic and agronomical damage to Chinese vineyards [15,16], specifically to the ‘Cabernet Sauvignon’ and ‘Chardonnay’ varieties.

*Xylotrechus arvicola* Olivier (Coleoptera: Cerambycidae), cerambycinae subfamily, is a destructive vineyard pest (*Vitis vinifera*) in the Iberian wine-producing areas [17]. *X. arvicola* females lay the eggs on crevices or under the rhytidome of the grapevine wood [18]. Most of the eggs hatch eight days after oviposition [19]. The fecundity of females and viability of eggs laid by *X. arvicola* females in the field last for a longer period than those kept in laboratory conditions [20]. *X. arvicola* eggs are white or cream coloured, quite homogeneous and elongated, with a length of around 1.8 mm and a width of approximately 0.7 mm on average. The larvae are legless and white [21]. After emerging from the egg, the larva penetrates inside the wood effortlessly, boring galleries within the plant’s wood [22]. The action of the larvae, together with the spread of wood fungi, causes direct damage (the larvae dig galleries that diminish the plant’s capacity to transport sap by reducing the vascular area) and indirect damage (fungal attack) [23]. Another problem is that *X. arvicola* adults are difficult to treat because they have a pattern of emergence which is considerably staggered in time [24]. Soria et al. [25] claimed that the emergence period takes place between late June and mid-July in vineyards of La Rioja and can continue until mid-August, while according to Moreno [21] this period goes from March until the end of July in vineyards of Valladolid (Castilla y Leon). Finally, Biurrun et al. [26] dated the emergence period in plantations of *Prunus spinosa* L in Navarre to be between 14 May and 26 August. Grapevine wood attacked by *X. arvicola* larvae is more sensitive to mechanical external factors in vineyards, such as strong winds, harvest weight (bunches of grapes produced by the vine), and vibration exerted by harvesting machines [27]. The most sensitive stages of this insect pest are the stages when they are adults and eggs. However, the larva can also be treated with insecticide during the first 24 h after hatching from the egg and before it bores into the vines, but once the *X. arvicola* larvae move into the wood, they cannot be reached with foliar-applied chemicals which cannot penetrate the vines [28].

The evaluation of insecticides against *X. arvicola* is challenging, as the conditions for a laboratory rearing that allows for a complete biological cycle have not yet been established. However, *X. arvicola* adults can be captured in the vineyards, and then larvae obtained from these adults can be reared on a semisynthetic diet for some time in the laboratory [29]. In this way, insecticides with different modes of action have been evaluated on eggs, larvae and adults [28], but active substances with low environmental impact are still needed. In fact, the most successful pesticide products in organic farming are based on *Bacillus thuringiensis* Berliner (Bt), and this insect group has not been previously evaluated with Bt, which could present opportunities for its environmentally friendly control.

*B. thuringiensis* is a gram-positive bacterium that produces pesticidal crystal proteins (Cry proteins) [30] and its effectiveness has been demonstrated against insects, nematodes, and other invertebrates [31,32,33]. The mode of action of Cry toxins on insects is summarised as follows: The proteins are synthesised as protoxins and then are cleaved by the midgut proteases to become active toxins after ingestion by the larvae [34,35]. Activated Cry toxins are inserted into the epithelial cell membrane of insects via binding to specific brush border receptors in order to elicit the formation of pores or to trigger a necrotic pathway, which eventually leads to the destruction of the midgut epithelial cells [36,37,38,39,40,41]. Finally, tissue damage and/or facilitated septicaemia led to the death of larvae [42,43].

Each Cry protein has a narrow insect toxicity spectrum, and most of those studied have been proteins with lepidopteran species as a target. However, several Cry proteins were reported to be toxic to a few coleopteran species or to be active against both orders [30]. Still, most of the genera, within the Coleoptera order, have not yet been evaluated with these Cry toxins [44].

Insects of the orders Lepidoptera, Coleoptera, and Diptera have been described as being highly sensitive to the toxicity of Cry proteins [45]. These include *Anticarsia gemmatalis* Hübner (Lepidoptera: Noctuidae) and *Chrysodeixis includens* Walker (Lepidoptera: Noctuidae) [46], *Mythimma separate* Walker (Lepidoptera: Noctuidae) [47], *Spodoptera frugiperda* (Lepidoptera: Noctuidae) and *Agrotis ipsilon* (Lepidoptera: Noctuidae) [48], *Ostrinia nubilalis* (Lepidoptera: Crambidae) [49], *Diabrotica barberi* Smith & Lawrence (Coleoptera: Chrysomelidae), *Diabrotica virgifera virgifera* LeConte (Coleoptera: Chrysomelidae) [50] and *Acanthoscelides obtectus* (Coleoptera: Chrisomelidae: Bruchinae) [51]. According to Van Frankenhuyzen [52], the Coleoptera is a broad and diverse insect order with numerous species that are crop pests, but only a few coleopteran species have been found to be susceptible to Bt toxins. Three-domain toxins belonging to different groups such as Cry1B, Cry1I, Cry3 and, Cry7 as well as other toxins, such as the jointly produced toxins Cry23A and Cry37A are toxic to different coleopteran species. These proteins’ lack of toxicity to vertebrates and to most other non-target organisms makes Bt toxins environmentally friendly when they are applied as biopesticides or in transgenic crops [32,45,53,54]. Yang et al. [47], stated that transgenic crops producing Bt toxins and have been globally adopted since 2010 kill the major target pests. Previous research [55,56,57] has described the effectiveness of the toxicity spectrum of Bt toxins against many pests, suggesting that only a few of them are appropriate to control a given pest. This is why it is also necessary to study the “lack of toxicity” of this type of protein against other organisms considered to be “non-object” since cross-order toxic activity against other insect orders could also arise [52,58,59].

As mentioned before, there are no previous references to the susceptibility of *X. arvicola* to Bt toxicity. So, our research was focused on evaluating for the first time the toxicological potential of different Cry proteins on coleopteran activity while under laboratory conditions. In this way, we will be able to gain knowledge of the effects of these proteins on the life parameters obtained in the laboratory.

## 2. Materials and Methods

### 2.1. X. arvicola Collection and Rearing

The *X. arvicola* neonate larvae (<24 h) used in the experiments were obtained from eggs laid by females after pairing with *X. arvicola* males captured in vineyards located in Gordoncillo (León, Castilla y León, Spain) (42°08′14.09″ N, 5°25′41.6″) and grown in the Protected Designation of Origin (PDO) ‘León’. PDO is a certificate to distinguish quality schemes for agricultural products and foodstuffs of a specific area (EC Reg. n. 1493/1999 published on 8 August 2009, OJC 187). The *X. arvicola* adults were captured using interception traps (Crosstrap^®^, Econex, Murcia, Spain) baited with ethanol as recommended by Rodríguez-González et al. [60]. The captured adults were paired (one female and one male) and put inside glass jars (80 mm in diameter and 100 mm high). The bottom of the jars was covered with filter paper on which substrates for oviposition (corrugated cardboard net rolls 120 × 40 mm) and drinking bowls (cotton soaked in a solution of 10% organic honey in distilled water) were placed. The oviposition substrates and drinking bowls were checked daily. The eggs were extracted and placed into Petri dishes (90 mm diameter), covered with aluminium foil to ensure it was dark, and the collection dates were noted. The *X. arvicola* stages before and after the application of treatments were kept in a chamber with a controlled temperature (24 ± 1 °C) and humidity (60 ± 5%) and subjected to a photoperiod of 16 h of light (luminous intensity of 1.000 µmol m^−2^s^−1^) and 8 h of darkness.

### 2.2. Cry Proteins Preparation

Cry proteins (Cry1Ba, Cry1Ia, Cry3Aa, Cry7Ab, and Cry23/37) were prepared from recombinant *B. thuringiensis* and *Escherichia coli* strains and solubilised in different carbonate buffers [51]. The protein concentrations were determined by the Bradford method [61]. Bovine Serum Albumin (BSA) was used as a standard. The purity of protein preparations was checked by 12% sodium dodecyl sulphate-polyacrylamide gel electrophoresis (SDS-PAGE), in which the main fragment corresponded to the molecular weight of each toxin for all the preparations. The toxins were lyophilised in powder for storage and suspended in a 0.1% solution of Tween 80 in distilled water to be used in the bioassays as reported by Rodríguez-González et al. [51]. Each solution was lyophilised to powder for storage.

### 2.3. Bioassays of Cry Proteins Sprayed on Artificial Diet to Rear X. arvicola Larvae

The bioassays were carried out using the newly hatched *X. arvicola* larvae (≤24 h) using the surface contamination method described by Ferré et al. [62]. A semi-synthetic diet [63] was used to perform the bioassays. This diet is adapted to the nutritional requirements of these insect species larvae [23,29], since it is the only artificial diet that ensures that the full biological cycle of some of the insects in the laboratory to be completed, given the difficulty of raising these insects outside their natural environment. The toxin, provided as a lyophilised powder was subsequently suspended in distilled water and 2 μg of each Cry toxin was applied to the surface of the diet placed in each well of the tray (2 cm^2^/well) (Greiner CELLSTAR^®^ 12 well plates, Sigma-Aldrich Chemie GmbH, Steinheim, Germany), carefully spread and allowed to dry under a laminar flow hood. The dose provided was 1 μg/cm^2^. As a control treatment, the diet in each well was contaminated with 100 µL of Na_2_CO_3_ 50 mM, a pH 10.5 buffer was used. Once dried, one larva was transferred to each well and confined by a cover (Greiner CELLSTAR^®^ 12 well plates). Thirty-six larvae distributed in three replicates of 12 well plates were used for each treatment. Prior to the Cry toxin applications, the surface of the diet was sterilised for 10 min under UV light. The larval mortality was scored 30 days after the application of the treatments, and larvae were considered dead if they did not have any reaction when prodded. In order to determine the effect of Cry toxins on the larval lifespan, the treated larvae were left in the well for 9 months, and the medium was changed every 30 days. The live larvae were counted at the end of the 9-month period.

The life parameters after the larval stage were determined by observing the development of treated larvae. Mortality and developmental time of each stage (the period of time that an individual was a larva, a prepupa, or a pupa) were recorded throughout the process. The prepupal stage started when the anterior part of the larva began to stiffen, and the posterior part performed rotary movements. The pupal (exact type) stage started when the different body parts of the future insect were easily recognisable, starting from when the antennae, mouthparts, legs, and wings were free or loose. The sex identification of the *X. arvicola* insects obtained in the laboratory was performed after the complete sclerotisation and melanisation of the adults when it was possible to distinguish body colours between males and females [21]. The larvae and adults were considered dead when they did not show movement after being touched several times with a brush.

### 2.4. Statistical Analysis

Cumulative survival data of the treated *X. arvicola* larvae during the 9 months were submitted to the Kaplan–Meier estimator and the functions obtained from each treatment were compared using the log—rank test (Mantel-Cox) (*p* < 0.05).

Differences in the duration of the last larval stages (prepupa and pupa) and adult lifespan (days of adult life) between treatments and between sexes were evaluated using a one-way ANOVA, while mean comparisons were performed using the posthoc LSD test (*p* < 0.05).

## 3. Results

### 3.1. Development of X. arvicola Larvae Treated with Cry Proteins during 9 Months

**Figure 1:**
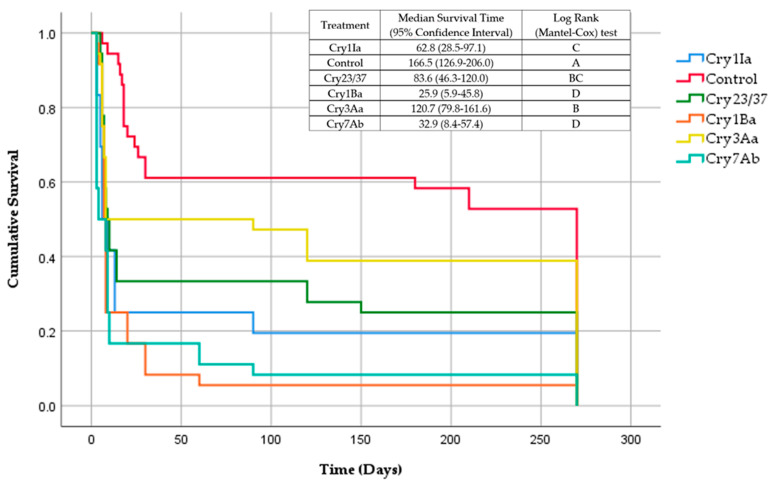
Kaplan–Meier cumulative survival curves for *Xylotrechus arvicola* larvae exposed to 1 µg/cm^2^ of Cry proteins applied to an artificial diet.

The *X. arvicola* larvae survival time was significantly affected (χ^2^ = 45.082; *p* < 0.001) by ingesting Cry proteins in their artificial diet (Figure 1). The larvae that were applied to the control treatment had a longer survival time (166.5 days) than the rest of the larvae to which Cry proteins were applied. By analysing these proteins and comparing them with each other, it was evident that the Cry 3Aa protein caused a significantly longer survival time in the larvae (120.7 days). At the other extreme, the Cry7Ab2 and Cry1Ba proteins significantly reduced the survival time of the larvae (32.9 and 25.9 days, respectively). 

### 3.2. Development Time of Pupal Stages and Adults’ Lifespan in Days after the Cry Protein Treatments

The larvae that did not complete the development (i.e., did not reach the adult stage) were recorded as dead. Significant differences (*p* = 0.647) in the developmental time of the prepupal and pupal stages of larvae (8 and 13 days as average values respectively) were not found among the treatments with the Cry proteins (Table 1).

The longevity of adults (Table 1) was affected by the treatment (*p* = 0.009) in a different way, so Cry23/37 extended their lifetime to 45 days, but Cy1Ia reduced this parameter to about half of the former (22 days). The control and Cry3Aa treatments provided intermediate values of about 38 days of life. To determine the sex influence on the different treatment responses, the lifespan was evaluated independently. No significant differences were detected when males and females were compared within each treatment (*p* > 0.05), this is probably due to the reduced number of insects analysed. Particular analyses of the males and females throughout all these treatments revealed that the lifespan of the females was not affected by the treatment applied. The treatment with Cry23/37 provided higher values for the lifespan of males and females, even though these values were not significantly different from those of the control treatment. This suggests that changes in adult lifespan were based on the effect of the treatment on male and female larvae.

Finally, the larval mortality of the control treatment was 25% after 241 days of development of the prepupal stage (which lasted 11 days longer), following 14 days of pupa stage. This accounts for about 266 days until their emergence as adults, and the 2 pupae that died increased the mortality to 29%. According to the days when the insects were adults a final duration of 302 days (about 10 months) as one whole lifespan was recorded. Regarding the Cry protein treatments, the relative number of emerged adults was lower for all treatments and especially for Cry1Ba, which only arrived at 33%. The whole lifespan reinforces this data showing the lowest duration (150 days).

All the adult insects obtained from the different treatments, (when they were paired with each other), were not fertile, since they were unable to lay eggs.

## 4. Discussion

The evaluation of active substances against coleopteran pests with a long and cryptic biological cycle (including *X. arvicola* larvae, which belongs to the Cerambycidae family) is a challenge. These laboratory tests were the initial steps in finding the proteins that are the most toxic for this pest, but setting them up precisely was a painstaking process. In regard to cerambycids, some authors pointed out that the critical phase for rearing in the laboratory appears to be their willingness to accept the diet in the first larval stage during their first month of life. For example, *Xylotrechus quadripes* (Coleoptera: Cerambycidae) in Visitpanich [64], or *Anoplophora macularia* (Coleoptera: Cerambycidae) in Lee and Lo [65]. Kosaka and Ogura [66] observed that neonate larvae of *Monochamus alternatus* (Hope) (Coleoptera: Cerambycidae) did not accept the diet in the first hours after being transferred to it, died during the first larval instars. We have successfully applied the bioassay protocol used with *X. arvicola* larvae to other treatments [22,28]. Our studies suggest that Cry proteins could minimise the damage caused by the *X. arvicola* larvae due to the significant reduction in larvae survival, particularly from Cry7Ab2 and Cry1Ba proteins (32.9 and 25.9 days, respectively), compared to the control (166.5 days). However, these data must be taken with care, since not all the larvae died when the proteins were applied. Thus, some larvae in the vineyard could also survive and produce a rapid resistance against these proteins if they are applied widely and repeatedly. Quick resistance evolution against Bt maize crops carrying the Cry1Ab protein was observed in the African maize stem borer, *Busseola fusca* (Fuller) (Lepidoptera: Noctuidae), which previously had not been fully controlled by Bt toxins [67]. Other studies described adverse effects on non-target and beneficial organisms [68,69,70]; resistance to Bt toxins that have emerged in target pests [71]; or problems with secondary (non-target) pests, as for example, with Bt cotton in China [72,73]. Having said that, these proteins could only be applied as bio-insecticides to vineyards during the emergence of *X. arvicola* adults (when first eggs and hatching young larvae can be exposed to these proteins), in order to avoid the appearance of rapid resistance against these toxins, and thus increase vine wood protection.

The average time of development at the prepupal stage was 11 days, whereas the pupal stage had an average of 14 days. This time of developmental at the pupal stage is very similar to that described by Wang et al. [74] in *Oemona hirta* (Coleoptera: Cerambycidae), in which the pupal stage lasted from 15 to 19 days, but a slightly lower than that described by Goodwin [14] in *Acalolepta vastator* Newman (Coleoptera: Cerambycidae) which varied from 19.6 days to 21.5 days. Gardiner [75] and Galford [76] described that rearing cerambycids larvae in the laboratory shortens the duration of the lifecycle in comparison to the field. However, the artificial diet used (SSI diet) for rearing *X. arvicola* larvae had enough quality to allow larvae to complete immature development and to obtain adults in the laboratory. In addition, the absence of egg-laying in the females obtained in this trial opens the possibility to study in future research the effect of the different Cry proteins on the fertility of adult insects. In previous works [23,29] *X. arvicola* insects were obtained in the laboratory using the same artificial diet. 

The total number of larvae that survived the 270 days of the trial in all treatments was 54, whereas the number of larvae that managed to complete their larval cycle and reach the adult stage was 46 insects, resulting in a total of 170 dead larvae or immature stages. Thus, the survival rate for all treatments was 21.3%. The survival percentages obtained by different authors in other cerambycids species were slightly above 50%. Wang et al. [74] scored a survival of 40% in *Oemona hirta* (Coleoptera: Cerambycidae) larvae, Dubois et al. [77] a survival of 41% in *Anoplophora glabripennis* (Coleoptera: Cerambycidae) larvae, and Lee and Lo [65] a survival of 31, 34 and 64% in *A. macularia* (Coleoptera: Cerambycidae) larvae. The adult lifespan (grouping the sexes together) ranged from 19.5 days to 39 days. When analysed by sex, the females lived between 26 and 42 days and the males between 20 and 48 days. Hanks [4] demonstrated that the cerambycids adult stage generally lasts between 36 and 53 days (similar values to those obtained in our experiments), depending on the sex and the rearing method employed. Results from other species of Subfamily Cerambycinae to which *X. arvicola* belongs, show life spans of adult insects’ adults to be 29 days for *Xylotrechus quadripes* [64].

## 5. Conclusions

Cry7Ab and Cry1Ba were the most effective in controlling *X. arvicola* larvae due to the significant reduction in larvae survival (32.9 and 25.9 days, respectively), and by causing serious alterations in the larvae during the remaining months of development. 

The developmental period of the prepupal and pupal stages was not affected by the previous ingestion of Cry proteins, so it can be deduced that the intake of these aforementioned proteins did not affect the final stages of the insect. 

The data presented suggest that these Cry proteins can be used as bioinsecticides against the larvae of this insect, by applying them only at the moment when the larvae hatch from the egg outside the grapevine wood (this would only be useful and justified if the economic threshold is exceeded), in order to avoid the appearance of rapid resistance against these toxins given the fact not all of the larvae were killed and thus, to increase vine wood protection.

## Figures and Tables

**Table 1:** Developmental time of pupal stages and adults’ lifespan in days after Cry protein treatments. The number of scored insects is given in parenthesis.

Treatments	Lifespan (Mean ± SE)		Adults’ Lifespan (Mean ± SE)					
	Prepupa	Pupa	Total	Male	Female	F	df	*p*
Control	10.8 ± 2.2 A (19) ^a^	13.8 ± 0.5 A (18) ^a^	36.2 ± 2.9 A (17) ^b^	37.9 ± 4.1 Aa (9) ^b^	34.2 ± 4.33 Aa (8) ^b^	0.375	1.15	0.550
Cry3Aa	7.6 ± 0.3 A (14)	13.9 ± 0.5 A (12)	39.6 ± 3.2 A (11)	39.0 ± 5.0 Aa (7)	40.7 ± 2.9 Aa (4)	0.060	1.9	0.811
Cry23/37	8.5 ± 0.7 A (9)	13.0 ± 0.7 A (9)	45.4 ± 4.0 A (8)	48.7 ± 4.3 Aa (4)	42.0 ± 7.0 Aa (4)	0.678	1.6	0.442
Cry1Ia	8.9 ± 1.4 A (7)	12.1 ± 1.4 A (7)	22.6 ± 3.9 B (5)	- (3)	-(2)	-	-	-
Cry7Ab	-(3)	- (3)	-(3)	- (1)	-(2)	-	-	-
Cry1Ba	-(2)	-(2)	-(2)	-(1)	-(1)	-	-	-
F	0.703	1.065	3.789	1.303	0.768			
df	3.45	3.42	5.48	2.17	2.17			
*p*	0.555	0.374	0.007	0.325	0.484			

Different capital letters within a column indicate significant differences (*p* ≤ 0.05; LSD test) among treatments for the same larval stage (prepupa and pupa). Different capital letters within the column indicate significant differences (*p* ≤ 0.05) among total insects; male insects (on the one hand) or among female insects (on the other hand) (*p* ≤ 0.05; LSD test). Different lowercase letters within this column indicate significant differences (*p* ≤ 0.05; LSD test) between sexes. ^a^ number of larvae in immature stages. ^b^ number of insects obtained. - no data available.

## Data Availability

Not applicable.
